# Current Evidence and Directions for Future Research in eHealth Physical Activity Interventions for Adults Affected by Cancer: Systematic Review

**DOI:** 10.2196/28852

**Published:** 2021-09-20

**Authors:** Manuel Ester, Maximilian Eisele, Amanda Wurz, Meghan H McDonough, Margaret McNeely, S Nicole Culos-Reed

**Affiliations:** 1 Faculty of Kinesiology University of Calgary Calgary, AB Canada; 2 Department of Physical Therapy University of Alberta Edmonton, AB Canada; 3 Department of Oncology University of Alberta Edmonton, AB Canada; 4 Rehabilitation Medicine Cross Cancer Institute Edmonton, AB Canada; 5 Department of Oncology Cummings School of Medicine University of Calgary Calgary, AB Canada; 6 Department of Psychosocial Resources Tom Baker Cancer Centre Cancer Care - Alberta Health Services Calgary, AB Canada

**Keywords:** eHealth, electronic health, mHealth, cancer, oncology, physical activity, exercise, systematic review, mobile phone

## Abstract

**Background:**

Physical activity (PA) interventions can increase PA and improve well-being among adults affected by cancer; however, most adults do not meet cancer-specific PA recommendations. Lack of time, facility access, and travel distances are barriers to participation in PA interventions. eHealth technologies may address some of these barriers, serving as a viable way to promote PA behavior change in this population. However, no review from July 2018 has synthesized available evidence across eHealth and cancer types or examined the use of behavioral theory and behavior change techniques (BCTs), leaving important gaps in knowledge.

**Objective:**

This review aims to provide a comprehensive, updated overview of evidence on eHealth PA interventions for adults with cancer by describing the current state of the literature, exploring associations between intervention characteristics and effectiveness, and identifying future research needs.

**Methods:**

MEDLINE, Embase, CINAHL, SportDiscus, Scopus, and CENTRAL were searched for eHealth PA interventions for adults affected by cancer. Study selection and data extraction were performed in duplicate, with consultation from the senior author (NCR). BCT coding, risk of bias, and completeness of reporting were performed using standardized tools. Results were summarized via narrative synthesis and harvest plots. Weight analyses were conducted to explore the associations between intervention characteristics and effectiveness.

**Results:**

A total of 71 articles (67 studies) involving 6655 participants (mean age 56.7 years, SD 8.2) were included. Nearly 50% (32/67) of the articles were published after July 2018. Significant postintervention PA increases were noted in 52% (35/67) of the studies, and PA maintenance was noted in 41% (5/12) of the studies that included a follow-up. Study duration, primary objectives, and eHealth modality (eg, websites, activity trackers, and SMS text messaging) varied widely. Social cognitive theory (23/67, 34%) was the most used theory. The mean number of BCTs used across the studies was 13.5 (SD 5.5), with self-monitoring, credible sources, and goal setting being used in >90% of studies. Weight analyses showed the greatest associations between increased PA levels and PA as a primary outcome (0.621), interventions using websites (0.656) or mobile apps (0.563), interventions integrating multiple behavioral theories (0.750), and interventions using BCTs of problem solving (0.657) and action planning (0.645). All studies had concerns with high risk of bias, mostly because of the risk of confounding, measurement bias, and incomplete reporting.

**Conclusions:**

A range of eHealth PA interventions may increase PA levels among adults affected by cancer, and specific components (eg, websites, use of theory, and action planning) may be linked to greater effectiveness. However, more work is needed to ascertain and optimize effectiveness, measure long-term effects, and address concerns with bias and incomplete reporting. This evidence is required to support arguments for integrating eHealth within PA promotion in oncology.

## Introduction

### Background

Physical activity (PA) can improve physical and psychosocial well-being among adults diagnosed with cancer. Benefits reported throughout the cancer trajectory (ie, from diagnosis onward) include enhanced physical functioning and quality of life, as well as reduced negative effects of cancer and treatment-related side effects [1]. Consequently, cancer-specific PA guidelines have been published, recommending at least 90 minutes of weekly moderate-intensity aerobic PA (note: before 2019, 150 minutes were recommended) and strength training for ≥2 days each week [2,3]. These guidelines have also been endorsed by leading cancer support organizations [4]. Despite this evidence, most adults diagnosed with cancer do not achieve the recommended PA levels [5].

Thus, developing and testing interventions to increase PA levels is a priority. As described in recent systematic reviews and meta-analyses, most interventions designed to enhance PA levels among individuals with cancer have been delivered face-to-face in fitness facilities, and findings suggest that such interventions can enhance physical and psychosocial well-being [6]. However, among adults diagnosed with cancer, barriers such as lack of time, limited access to facilities, and travel distances can hinder participation in face-to-face PA interventions [7]. Barriers to PA have been exacerbated during the COVID-19 pandemic, with most face-to-face PA opportunities being limited or canceled and adults with cancer reporting decreased PA and increased sedentary time [8].

eHealth technologies, including telephones, websites, email, and mobile health (mHealth) technologies (eg, SMS text messaging, smartphones, wearable technology, and apps) may be useful to address some of these barriers to PA and reach a wider audience of adults living with cancer [9-11]. The prevalence of and preference for using eHealth is increasing rapidly among adults with cancer, with the National Cancer Institute prioritizing research into the effective use of eHealth in the context of PA promotion for adults with cancer [12-14]. Reviews summarizing the effects of eHealth to promote PA in adults with cancer suggest that technology-supported PA interventions may enhance PA levels and health-related quality of life and decrease fatigue [15-19]. Notwithstanding the evidence to date, important gaps in knowledge remain. First, only studies published before July 2018 have been reviewed. As the field of eHealth PA interventions is rapidly growing and evolving, an update is needed. Second, reviews have had limited scope with regard to study design (eg, randomized controlled trials [RCTs] only [18]), population (eg, women with breast cancer only [19]), and technology components (eg, activity trackers or mHealth only [16,17,19]). Expanding eligibility criteria to include various study designs, cancer types, and the full range of eHealth technologies is required to provide a more comprehensive overview of the effects of eHealth PA interventions in oncology. Finally, despite evidence supporting the role of behavior change techniques (BCTs) and theories (eg, theory of planned behavior) in PA interventions, the integration of BCTs and theory with eHealth PA interventions has received limited attention [15,18,20-22]. Roberts et al [15] examined the use of theory and BCTs for 15 eHealth PA interventions published before November 2016, whereas Kiss et al [18] coded BCTs for 16 interventions, many of which were duplicates from Roberts et al [15], published before July 2018.

### Objectives

Thus, the purpose of this review is to summarize evidence on the use of eHealth to support PA behavior change among adults diagnosed with cancer. The specific objectives are to (1) describe the current state of the literature on the effectiveness of eHealth in supporting PA behavior change (pre- to postintervention and follow-ups, where available), (2) explore intervention characteristics that may promote PA behavior change (eg, eHealth components, use of theory, and BCTs), and (3) identify research needs for future work.

## Methods

The review protocol was registered prospectively via PROSPERO (International Prospective Register of Systematic Reviews): CRD42020162181. Reporting of the results follows the PRISMA (Preferred Reporting Items for Systematic Reviews and Meta-Analyses) guidelines for systematic reviews [23].

### Search Strategy

For identifying relevant studies, a search strategy covering the major topics of health technology, cancer, and PA was developed in MEDLINE (R) using existing reviews to guide the selection of search terms. It was then refined, finalized, and translated to the other databases used herein with the help of a university librarian (Table S1 in Multimedia Appendix 1). MEDLINE (R) and Epub Ahead of Print, In-Process and Other Non-Indexed Citations and Daily (OVID), Embase (OVID), CENTRAL (OVID), CINAHL (EBSCO), Sport Discus (EBSCO), and Scopus were searched from database inception through to December 18, 2019. This search was updated on January 7, 2021.

### Eligibility Criteria

To be included, articles had to (1) comprise adult participants aged ≥18 years diagnosed with cancer, (2) evaluate a PA intervention that used technology (mobile app, SMS text messages, wearable activity tracker, website, email, or other eHealth) as an active component in the intervention to support behavior change, (3) measure and report on PA levels (objectively or subjectively), (4) be published in English, and (5) be published in a peer-reviewed journal (conference abstracts and gray literature were not included). Articles were excluded if they (1) involved adults whose only cancer diagnosis occurred during childhood, adults without a history of cancer, or caregivers; (2) used telephone contact as the only technology component in the intervention; (3) used technology for the measurement of outcomes only (eg, accelerometer for PA measurement pre- or postintervention); (4) lacked a PA intervention (eg, observational study of PA behavior); (5) reported ongoing trials without full results being available (ie, protocols); and (6) the full text was unavailable. Interventions could be either partially supervised (ie, some human contact) or unsupervised (ie, entirely automated), and the amount of technology use within interventions was not quantified.

### Study Selection

After importing all search results into EndNote X9.2 (Clarivate Analytics), the first author conducted automatic and subsequent manual deduplication. Unique articles were exported to Rayyan (Rayyan Systems) for screening according to the eligibility criteria [24]. Title and abstract screening were conducted concurrently by the first author by removing all articles that did not meet the criteria. Articles with titles and abstracts that lacked enough information to make a decision were carried forward to the full-text screening stage. Full texts of the remaining articles were obtained and screened independently by the first (ME) and second authors (MME), who recorded their decisions as well as reasons for exclusion where applicable. The 2 authors then met to discuss the decisions and resolve disagreements based on additional reviews of the articles. Disagreements that could not be resolved directly were resolved via discussion with the senior author (NCR) to yield the final list of included articles.

### Data Extraction

Before data extraction, a standardized data extraction table was developed and refined using 3 test articles. The final data extraction table included (1) participant information (age, cancer diagnosis, and eligibility criteria), (2) study design (timing, eligibility and recruitment rates, and recruitment methods), (3) intervention details (groups, objectives, duration, active components, technology integration, BCTs according to the Michie behavior change taxonomy comprising 93 BCTs across 16 categories [25], and use of theory), (4) outcomes (participant numbers, demographics, primary and secondary outcomes, PA-related outcomes, adherence or completion to intervention, and technology use), and (5) additional factors (key findings, challenges, and limitations). It was decided that theory would be recorded only when explicitly described in the included studies. Data were then extracted independently by the first (ME) and second authors (MME), with each author being responsible for half the number of articles. For confirming the reliability of the extraction, 5 random articles were exchanged between authors, extracted a second time, and the data were compared between extractions. Because of minor discrepancies, coding of BCTs was repeated for all articles, and discussions were held between the first and second authors to reach a consensus. The authors did not complete BCT coder training before BCT coding. No other discrepancies were noted. Any missing information was denoted using the phrase *not reported* in the data extraction table. Attempts were made to fill in missing information via protocol papers and other related publications for each study. The authors of the included articles were not contacted directly for additional information.

### Risk of Bias and Completeness of Reporting

The Cochrane risk of bias (RoB) tool (RoB-2) was used for multiarm interventions, which included evaluations for RoB in five domains: (1) randomization, (2) deviation from the intended intervention, (3) missing outcome data, (4) measurement of the outcome, and (5) selection of reported results [26]. The ROBINS-I (RoB in nonrandomized studies of interventions) tool, which evaluates bias across seven domains: (1) confounding, (2) participant selection, (3) classification of intervention, (4) deviation from intended intervention, (5) missing data, (6) outcome measurement, and (7) selection of reported results was used for single-arm designs [27]. An overall RoB was given according to the highest RoB rating in any domain for each study. For example, a study with high RoB in domain 1 and low RoB across all other domains received a high overall RoB rating. The completeness of reporting was evaluated using the CONSORT (Consolidated Standards of Reporting Trials)–eHealth checklist, with items assessed as reported, not reported, or not applicable [28]. The completeness of reporting score was calculated for each article as the percentage of applicable items that were reported. These assessments were performed independently by the first (ME) and second authors (MME). Verification was performed by exchanging 5 random articles between authors for repeat assessment, and no discrepancies were documented.

### Data Synthesis and Analysis

To summarize the data extracted from each article, descriptive statistics were calculated for participant demographics, adherence, and completion. Intervention details were categorized and summarized, whereas results were converted to standardized metrics where possible to enable comparison across studies. Because of the substantial heterogeneity of the studies with regards to population, intervention, comparison, and outcome, meta-analyses were not performed. Instead, extracted data across studies were summarized using narrative synthesis techniques, and summary tables were presented [29]. Harvest plots were created to provide a visual summary of study effects on PA outcomes, including PA levels directly postintervention and PA maintenance at follow-up, providing an overview of intervention effectiveness on PA levels [30]. Following recommendations, harvest plots were prepared with studies grouped according to the statistical significance of their PA outcomes (PA increase, PA decrease, or no change) [30]. Bar heights were used to distinguish between RCTs (high) and other study designs (low), whereas shading was used to specify how PA was measured (subjective, objective, or both). For addressing objective 2, weight analyses were conducted to explore associations between independent variables (intervention characteristics: use of supervised elements, various types of eHealth, theory, and BCTs) and the dependent variable (PA levels) [31]. Weight was calculated for each independent or dependent variable pair by dividing the number of studies featuring each independent variable and reporting a significant improvement in the dependent variable by the total number of studies featuring the independent variable. Weights range from 0-1, with a higher value indicating a stronger association between the independent variable and significant changes in PA levels. Weights are presented to three decimal places and are equivalent to percentages (ie, 0.123 could also be read as 12.3%). The weight for each independent or dependent variable pairing was then compared with the overall weight for all studies to explore if the presence of certain intervention characteristics was associated with a higher weight (ie, more often linked with significant changes in PA levels). For continuous independent variables (duration and number of BCTs used), studies were grouped according to the mean value (greater than or less than the mean). For BCTs, weights were only calculated for the most common BCTs or BCT categories (ie, used in at least 50% of interventions) to minimize the introduction of further bias when calculating weights using only a small number of independent or dependent variable pairs [31].

## Results

### Study Selection

**Figure 1:**
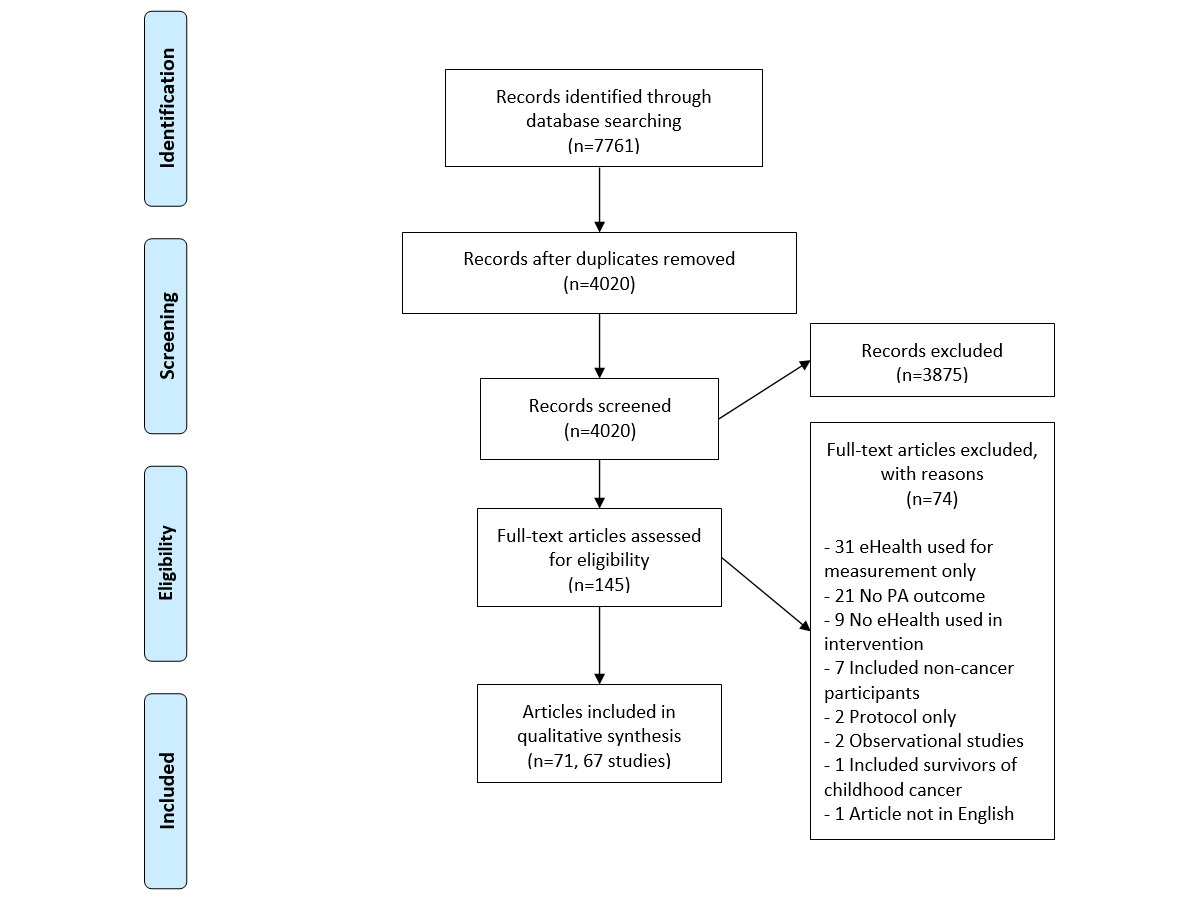
PRISMA (Preferred Reporting Items for Systematic Reviews and Meta-Analyses) flow diagram of article selection. PA: physical activity.

After deduplication, 4022 citations were screened at the title or abstract level; of the 4022 citations, 3873 (96.29%) were removed as they did not meet the eligibility criteria. During full-text screening, the agreement between the first 2 authors on the 145 articles was 82.1%, with decisions for articles where no agreement was reached (26/145, 17.9%) being resolved via discussion with the senior author (NCR). Of the 145 articles, 74 (51.0%) articles were excluded during full-text screening, and, overall, 71 (49.0%) articles representing 67 unique studies were included [32-102]. Figure 1 presents an overview of the study selection, with reasons for article exclusion. The remainder of the results are presented according to the number of unique studies (n=67). Tables 1 and 2 provide more information on each of the included studies and their respective PA interventions.

**Table 1:** Overview of study type, participant characteristics, and outcomes^a^.

Reference	Study type	Participant characteristics	Study outcomes
Mayo et al [32]	RCT^b^	Cancer: advanced mixed Treatment: any n=26 Age (years), median: 57.0 Female (%): 46	Primary outcome: Fatigue Secondary outcome: PA^c^, physical and psychosocial
Maxwell-Smith et al [33]	RCT	Cancer: mixed Treatment: off n=68 Age (years), mean (SD): 64.1 (7.9) Female (%): 50 Caucasian (%): 97	Primary outcome: PA Secondary outcome: Sedentary and physical
Park et al [34]	RCT	Cancer: advanced prostate Treatment: any n=21 Age (years), median: 66.5 Female (%): 0	Primary outcome: Psychosocial Secondary outcome: PA
Gomersall et al [35]	RCT	Cancer: any Treatment: any n=36 Age (years), mean (SD): 64.8 (9.6) Female (%): 36	Primary outcome: Feasibility Secondary outcome: PA and sedentary
Gehring et al [36]	RCT	Cancer: brain Treatment: off n=34 Age (years): 48.0 Female (%): 56	Primary outcome: Feasibility Secondary outcome: PA, physical, and psychosocial
Singh et al [37]	RCT	Cancer: breast Treatment: any n=52 Age (years), mean (SD): 51.2 (9.0) Female (%): 100	Primary outcome: PA Secondary outcome: Feasibility
Buscemi et al [38]	RCT	Cancer: breast Treatment: any n=80 Age (years), mean (SD): 52.5 (11.4) Female (%): 100 Caucasian (%): 0	Primary outcome: PA, Nutrition
Chapman et al [39]	RCT	Cancer: breast Treatment: off n=101 Age (years), mean (SD): 59.1 (8.2) Female (%): 100 Caucasian (%): 93	Primary outcome: PA Secondary outcome: Psychosocial
Fazzino et al [40]	RCT	Cancer: breast Treatment: off n=142 Age (years), mean (SD): 58.6 (8.0) Female (%): 100 Caucasian (%): 97	Primary outcome: Physical Secondary outcome: PA
Hartman et al [41]	RCT	Cancer: breast Treatment: off n=42 Age (years), mean (SD): 57.9 (11.3) Female (%): 100 Caucasian (%): 81	Primary outcome: Psychosocial Secondary outcome: PA
Hatchett et al [42]	RCT	Cancer: breast Treatment: off n=74 Female (%:) 100 Caucasian (%): 95	Primary outcome: PA Secondary outcome: Sedentary
Lynch et al [43,44]	RCT	Cancer: breast Treatment: off n=83 Age (years), mean (SD): 61.6 (6.4) Female (%): 100	Primary outcome: PA Secondary outcome: Sedentary
McNeil et al [45]	RCT	Cancer: breast Treatment: off n=45 Age (years), mean (SD): 58.7 (9.3) Female (%): 100 Caucasian (%): 80	Primary outcome: PA Secondary outcome: Sedentary, Physical, Psychosocial
Park et al [46]	RCT	Cancer: breast Treatment: off n=356 Age (years), mean (SD): 50.3 (9.5) Female (%): 100	Primary outcome: PA
Paxton et al [47]	RCT	Cancer: breast Treatment: off n=71 Age (years), mean (SD): 52.2 (8.5) Female (%): 100	Primary outcome: PA Secondary outcome: Nutrition
Pope et al [48]	RCT	Cancer: breast Treatment: off n=30 Female (%): 100 Caucasian (%): 97	Primary outcome: PA Secondary outcome: Sedentary, physical, and psychosocial
Short et al [49]	RCT	Cancer: breast Treatment: off n=492 Age (years), mean (SD): 55.1 (9.7) Female (%): 100	Primary outcome: Feasibility Secondary outcome: PA
Uhm et al [50]	RCT	Cancer: breast Treatment: off n=356 Age (years), mean (SD): 50.3 (9.5) Female (%): 100	Primary outcome: PA
Weiner et al [51]	RCT	Cancer: breast Treatment: off n=87 Age (years): 57.2 Female (%): 100 % Caucasian (%): 82	Primary outcome: PA Secondary outcome: Sedentary
Allicock et al [52]	RCT	Cancer: breast Treatment: off n=22 Age (years), mean (SD): 52.2 (9.2) Female (%): 100 Caucasian (%): 0	Primary outcome: Feasibility Secondary outcome: PA, Nutrition
Gokal et al [53]	RCT	Cancer: breast Treatment: on n=50 Age (years): 52.2 Female (%): 100	Primary outcome: Psychosocial Secondary outcome: PA
Van Blarigan et al [54]	RCT	Cancer: colorectal Treatment: off n=41 Age (years), mean (SD): 54.0 (11.0) Female (%): 59 Caucasian (%): 73	Primary outcome: Feasibility Secondary outcome: PA
Haggerty et al [55]	RCT	Cancer: endometrial Treatment: off n=41 Age (years), mean (SD): 59.7 (8.7) Female (%): 100 Caucasian (%): 78	Primary outcome: Physical Secondary outcome: PA
Chow et al [56]	RCT	Cancer: leukemia lymphoma Treatment: off n=41 Age (years): 45.1 Female (%): 49 Caucasian (%): 78	Primary outcome: Feasibility Secondary outcome: PA, Physical, psychosocial, and nutrition
Edbrooke et al [57]	RCT	Cancer: lung Treatment: on n=80 Age (years), mean (SD): 63.1 (12.3) Female (%): 44	Primary outcome: Physical Secondary outcome: PA, Psychosocial
Cox et al [58]	RCT	Cancer: mixed Treatment: any n=37 Age (years): 59.7 Female (%): 0 Caucasian (%): 84	Primary outcome: Physical Secondary outcome: PA
Forbes et al [59]	RCT	Cancer: mixed Treatment: any n=95 Age (years), mean (SD): 65.1 (8.5) Female (%): 56 Caucasian (%): 99	Primary outcome: Feasibility Secondary outcome: PA, Psychosocial
Golsteijn et al [60]	RCT	Cancer: mixed Treatment: any n=478 Age (years): 66.5 Female (%): 13	Primary outcome: PA Secondary outcome: Physical, Fatigue, Psychosocial
Ormel et al [61]	RCT	Cancer: mixed Treatment: any n=32 Age (years): 33.6 Female (%): 13	Primary outcome: Feasibility Secondary outcome: PA
Webb et al [62,63]	RCT	Cancer: mixed Treatment: any n=207 Female (%): 74 Caucasian (%): 97	Primary outcome: PA Secondary outcome: Psychosocial
Bantum et al [64]	RCT	Cancer: mixed Treatment: off n=352 Age (years), mean (SD): 50.9 (11.0) Female (%): 82 Caucasian (%): 87	Primary outcome: Fatigue Secondary outcome: PA, psychosocial, and nutrition
Frensham et al [65,66]	RCT	Cancer: mixed Treatment: off n=91 Age (years), mean (SD): 65.8 (9.4) Female (%): 52 Caucasian (%): 96	Primary outcome: PA Secondary outcome: Physical and psychosocial
Gell et al [67]	RCT	Cancer: mixed Treatment: off n=66 Age (years), mean (SD): 61.4 (9.0) Female (%): 83 Caucasian (%): 99	Primary outcome: Feasibility Secondary outcome: PA
Kanera et al [68,69]	RCT	Cancer: mixed Treatment: off n=462 Age (years), mean (SD): 55.9 (11.4) Female (%): 80	Primary outcome: PA Secondary outcome: Nutrition
Mayer et al [70]	RCT	Cancer: mixed Treatment: off n=284 Age (years), mean (SD): 58.6 (14.0) Female (%): 52 Caucasian (%): 89	Primary outcome: PA
Park et al [71]	RCT	Cancer: mixed Treatment: off n=162 Age (years), mean (SD): 51.8 (8.0) Female (%): 88	Primary outcome: PA Secondary outcome: Psychosocial
Valle et al [72]	RCT	Cancer: mixed Treatment: off n=86 Age (years): 31.7 Female (%): 91 Caucasian (%): 91	Primary outcome: Feasibility Secondary outcome: PA, physical, and psychosocial
Rabin et al [73]	RCT	Cancer: mixed Treatment: off n=18 Age (years), mean (SD): 32.2 (5.6) Female (%): 56 Caucasian (%): 84	Primary outcome: PA Secondary outcome: Feasibility, fatigue, and psychosocial
Robertson et al [74]	RCT	Cancer: mixed Treatment: off n=78 Age (years), mean (SD): 55.1 (13.5) Female (%): 91 Caucasian (%): 80	Primary outcome: Feasibility Secondary outcome: PA and psychosocial
Yun et al [75]	RCT	Cancer: mixed Treatment: off n=394 Age (years), mean (SD): 54.0 (11.0) Female (%): 61	Primary outcome: PA Secondary outcome: Physical and psychosocial
Shang et al [76]	RCT	Cancer: mixed Treatment: on n=126 Age (years), mean (SD): 60.2 (10.6) Female (%): 39 Caucasian (%): 81	Primary outcome: PA
Villaron et al [77]	RCT	Cancer: mixed Treatment: on Female (%): 0	Primary outcome: PA Secondary outcome: Fatigue and psychosocial
Chan et al [78]	RCT	Cancer: prostate Treatment: any n=202 Age (years), median: 70 Female (%): 0 Caucasian (%): 93	Primary outcome: Feasibility Secondary outcome: PA and nutrition
Kenfield et al [79]	RCT	Cancer: prostate Treatment: off n=78 Age (years), median: 65 Female (%): 0 Caucasian (%): 78	Primary outcome: Feasibility Secondary outcome: PA and psychosocial
Alibhai et al [80]	RCT	Cancer: prostate Treatment: on n=53 Age (years): 70.0 Female (%): 0 Caucasian (%): 72	Primary outcome: Feasibility Secondary outcome: PA, physical, and psychosocial
Bade et al [81]	Other	Cancer: advanced lung Treatment: any n=37 Age (years), mean (SD): 66.4 (8.6) Female (%): 30	Primary outcome: PA
Naito et al [82]	Other	Cancer: advanced mixed Treatment: on n=30 Age (years), median: 75 Female (%): 33	Primary outcome: Feasibility Secondary outcome: PA
Befort et al [83]	Other	Cancer: breast Treatment: off n=34 Age (years), mean (SD): 58.9 (7.8) Female (%): 100 Caucasian (%): 97	Primary outcome: Physical Secondary outcome: Feasibility, PA, and nutrition
Nápoles et al [84]	Other	Cancer: breast Treatment: off n=23 Age (years), mean (SD): 55.8 (13.1) Female (%): 100	Primary outcome: Feasibility Secondary outcome: PA, fatigue, and psychosocial
Pope et al [85]	Other	Cancer: breast Treatment: off n=10 Age (years), mean (SD): 45.8 (10.2) Female (%): 100 Caucasian (%): 90	Primary outcome: Feasibility Secondary outcome: PA, physical, and psychosocial
Spark et al [86]	Other	Cancer: breast Treatment: off n=29 Age (years), mean (SD): 54.9 (8.8) Female (%): 100 Caucasian (%): 97	Primary outcome: Feasibility Secondary outcome: PA, physical, and nutrition
Wilson et al [87]	Other	Cancer: breast Treatment: off n=22 Age (years): 55.0 Female (%): 100 Caucasian (%): 0	Primary outcome: Feasibility Secondary outcome: PA and physical
Chung et al [88]	Other	Cancer: breast Treatment: off n=54 Age (years), mean (SD): 44.5 (6.40) Female (%): 100 Caucasian (%): 0	Primary outcome: PA Secondary outcome: Psychosocial
Nyrop et al [89]	Other	Cancer: breast Treatment: on n=100 Age (years), mean (SD): 48.3 (9.4) Female (%): 100 Caucasian (%): 69	Primary outcome: PA
Cairo et al [90]	Other	Cancer: breast Treatment: on n=127 Age (years), mean (SD): 54.1 (9.0) Female (%): 100 Caucasian (%): 95	Primary outcome: PA, Nutrition Secondary outcome: Fatigue and psychosocial
Cheong et al [91]	Other	Cancer: colorectal Treatment: on n=75 Age (years), mean (SD): 58.3 (11.7) Female (%): 41	Primary outcome: PA Secondary outcome: Feasibility, physical, and psychosocial
Groen et al [92]	Other	Cancer: lung Treatment: any n=34 Age (years), mean (SD): 59.6 (8.4) Female (%): 47	Primary outcome: Feasibility Secondary outcome: PA and psychosocial
Hong et al [93]	Other	Cancer: mixed Treatment: any n=26 Age (years), median: 69 Female (%): 69 Caucasian (%): 73	Primary outcome: Psychosocial Secondary outcome: Feasibility and PA
McCarroll et al [94]	Other	Cancer: mixed Treatment: any n=50 Age (years), mean (SD): 58.4 (10.3) Female (%): 100 Caucasian (%): 88	Primary outcome: Feasibility Secondary outcome: PA, physical, psychosocial, and nutrition
MacDonald et al [95]	Other	Cancer: mixed Treatment: any n=35 Age (years), mean (SD): 55.0 (15.9) Female (%): 63	Primary outcome: Feasibility Secondary outcome: PA, physical, and psychosocial
Gell et al [96]	Other	Cancer: mixed Treatment: off n=24 Age (years), mean (SD): 57.5 (10.4) Female (%): 83 Caucasian (%): 92	Primary outcome: PA Secondary outcome: Feasibility
Puszkiewicz et al [97]	Other	Cancer: mixed Treatment: off n=45 Age (years), mean (SD): 64.6 (13.4) Female (%): 51	Primary outcome: Feasibility Secondary outcome: PA, fatigue, and psychosocial
Short et al [98]	Other	Cancer: mixed Treatment: off n=12 Age (years), mean (SD): 56.0 (11.1) Female (%): 60	Primary outcome: Feasibility Secondary outcome: PA
Abbott et al [99]	Other	Cancer: mixed Treatment: on n=39 Age (years): 57.0 Female (%): 69 Caucasian (%): 97	Primary outcome: Fatigue Secondary outcome: PA
Javaheri et al [100]	Other	Cancer: mixed Treatment: on n=21 Age (years), median: 56 Female (%): 86	Primary outcome: Feasibility Secondary outcome: PA and psychosocial
Zhang et al [101]	Other	Cancer: ovarian Treatment: any n=10 Age (years), median: 63 Female (%): 100 Caucasian (%): 100	Primary outcome: Feasibility Secondary outcome: PA
Trinh et al [102]	Other	Cancer: prostate Treatment: on n=46 Age (years), mean (SD): 73.2 (7.3) Female (%): 0 Caucasian (%): 80	Primary outcome: Feasibility Secondary outcome: PA, sedentary, and psychosocial

^a^Studies were sorted by study type, cancer type, and treatment. Of note, some articles did not report certain participant characteristics, such as ethnicity or age.

^b^RCT: randomized controlled trial.

^c^PA: physical activity.

**Table 2:** Overview of intervention duration, supervision, physical activity measure, delivery components, use of theory, and behavior change techniques^a^.

Reference	Intervention design	PA^b^	Delivery			Theory		Total number of BCT^c^/ number of BCT categories covered
			eHealth	Additional				
Mayo et al [32]	Duration (weeks): 16; follow-up (weeks): 24; no supervision	Objective	WAT^d^ and phone	Exercise goal or program and phone counseling	Theory on etiology and treatment of cancer-related fatigue		13/8	
Maxwell-Smith et al [33]	Duration (weeks): 12; partial supervision	Objective	Website, WAT, and SMS text messaging	Print materials, phone counseling, in-person counseling, and group interaction	HAPA^e^		15/9	
Park et al [34]	Duration (weeks): 8; partial supervision	Subjective and objective	SMS text messaging	PA log, print materials, and in-person counseling	SDT^f^		14/9	
Gomersall et al [35]	Duration (weeks): 12; partial supervision	Subjective and objective	SMS text messaging	Exercise goal or program and in-person counseling	SCT^g^		16/10	
Gehring et al [36]	Duration (weeks): 26; partial supervision	Subjective	Website, WAT, and email	PA log, print materials, and in-person counseling	None		9/5	
Singh et al [37]	Duration (weeks): 12; partial supervision	Subjective and objective	Website and WAT	Print materials and in-person counseling	TPB^h^		7/5	
Buscemi et al [38]	Duration (weeks): 6; no supervision	Subjective	SMS text messaging and mobile app	Phone counseling	None		6/5	
Chapman et al [39]	Duration (weeks): 4; follow-up (weeks): 12; no supervision	Subjective	Website	None	TTM^i^		6/2	
Fazzino et al [40]	Duration (weeks): 52; no supervision	Subjective and objective	WAT and phone	Exercise goal or program, PA log, phone counseling, group interaction, and DVD	SCT		11/8	
Hartman et al [41]	Duration (weeks): 12; partial supervision	Objective	Website, WAT, email, and phone	Exercise goal or program, phone counseling, and in-person counseling	TTM and SCT		13/8	
Hatchett et al [42]	Duration (weeks): 12; no supervision	Subjective	Email	None	SCT		16/10	
Lynch et al [43,44]	Duration (weeks): 12; partial supervision	Objective	Website and WAT	Exercise goal or program, print materials, phone counseling, and in-person counseling	Behavior change strategies		16/8	
McNeil et al [45]	Duration (weeks): 12; follow-up (weeks): 24; no supervision	Objective	WAT, email, and phone	PA log and phone counseling	None		13/7	
Park et al [46]	Duration (weeks): 12; no supervision	Subjective	WAT and mobile app	Exercise goal or program	None		11/7	
Paxton et al [47]	Duration (weeks): 12; no supervision	Subjective	Website and email	Exercise goal or program	SCT, TTM, goal-setting theory, and social marketing		24/12	
Pope et al [48]	Duration (weeks): 10; no supervision	Objective	Website and WAT	Exercise goal or program and group interaction	SCT		21/12	
Short et al [49]	Duration (weeks): 12; no supervision	Subjective	Website and email	None	SCT		18/11	
Uhm et al [50]	Duration (weeks): 12; no supervision	Subjective	WAT and mobile app	Exercise goal or program	None		14/9	
Weiner et al [51]	Duration (weeks): 12; no supervision	Objective	WAT, email, and phone	Phone counseling and in-person counseling	SCT		17/10	
Allicock et al [52]	Duration (weeks): 4; no supervision	Subjective and objective	SMS text messaging and mobile app	PA log and print materials	SCT		9/8	
Gokal et al [53]	Duration (weeks): 12; no supervision	Subjective and objective	WAT	PA log	TPB		12/8	
Van Blarigan et al [54]	Duration (weeks): 12; partial supervision	Objective	Website, WAT, and SMS text messaging	Print materials	TPB		12/9	
Haggerty et al [55]	Duration (weeks): 24; no supervision	Subjective	Website, SMS text messaging, and phone	Exercise goal or program and PA log	None		15/8	
Chow et al [56]	Duration (weeks): 16; no supervision	Subjective and objective	WAT, email, SMS text messaging, mobile app, and phone	Phone counseling and group interaction	SDT		12/6	
Edbrooke et al [57]	Duration (weeks): 8; follow-up (weeks): 26; partial supervision	Objective	WAT, SMS text messaging, and phone	Exercise goal or program, PA log, phone counseling, in-person counseling, and DVD	None		18/11	
Cox et al [58]	Duration (weeks): 26; no supervision	Subjective and objective	Website, WAT, email, and phone	Exercise goal or program and group interaction	SCT and TTM		8/6	
Forbes et al [59]	Duration (weeks): 9; no supervision	Subjective	Website and email	None	Unspecified *theory-based*		16/10	
Golsteijn et al [60]	Duration (weeks): 26; follow-up (weeks): 16; no supervision	Subjective and objective	Website and WAT	None	SCT, TTM, HAPA, I-Change model, and health belief model		16/10	
Ormel et al [61]	Duration (weeks): 12; no supervision	Subjective	Email, mobile app, and phone	PA log and phone counseling	None		9/7	
Webb et al [62,63]	Duration (weeks): 12; follow-up (weeks): 24; no supervision	Subjective	Website	PA log, print materials, group interaction, and DVD	SCT and TPB		24/12	
Bantum et al [64]	Duration (weeks): 6; no supervision	Subjective	Website and phone	Print materials and group interaction	None		18/10	
Frensham et al [65,66]	Duration (weeks): 12; follow-up (weeks): 24; no supervision	Objective	Website and WAT	Exercise goal or program, PA log, and group interaction	SCT		9/5	
Gell et al [67]	Duration (weeks): 8; partial supervision	Objective	Website, WAT, SMS text messaging, and phone	In-person counseling	SCT		11/6	
Kanera et al [68,69]	Duration (weeks): 26; no supervision	Subjective	Website and email	None	SCT		14/7	
Mayer et al [70]	Duration (weeks): 26; no supervision	Subjective	WAT, mobile app, and phone	Print materials, phone counseling, and group interaction	SDT		16/10	
Park et al [71]	Duration (weeks): 4; no supervision	Subjective	WAT	Exercise goal or program, PA log, and DVD	None		10/8	
Valle et al [72]	Duration (weeks): 12; no supervision	Subjective	Website	Exercise goal or program, PA log, and group interaction	SCT		19/11	
Rabin et al [73]	Duration (weeks): 12; no supervision	Subjective	Website and email	None	SCT and TTM		14/9	
Robertson et al [74]	Duration (weeks): 4; no supervision	Subjective and objective	Website, WAT, SMS text messaging, and mobile app	None	SDT, behavior change wheel, and motivational interviewing		23/14	
Yun et al [75]	Duration (weeks): 12; follow-up (weeks): 24; partial supervision	Subjective	Website and phone	Print materials, phone counseling, and in-person counseling	None		10/6	
Shang et al [76]	Duration (weeks): 12; no supervision	Subjective and objective	WAT and phone	Exercise goal or program, PA log, and phone counseling	None		14/8	
Villaron et al [77]	Duration (weeks): 8; no supervision	Objective	WAT and SMS text messaging	Print materials	None		11/8	
Chan et al [78]	Duration (weeks): 12; follow-up (weeks): 24; no supervision	Subjective	Website, WAT, SMS text messaging, and phone	Phone counseling	SCT		10/8	
Kenfield et al [79]	Duration (weeks): 12; no supervision	Subjective and objective	Website, WAT, email, and SMS text messaging	Exercise goal or program	TPB		18/10	
Alibhai et al [80]	Duration (weeks): 26; partial supervision	Subjective and objective	WAT, mobile app, and phone	Exercise goal or program, phone counseling, and group interaction	None		11/9	
Bade et al [81]	Duration (weeks): 4; no supervision	Objective	WAT, SMS text messaging, and phone	Phone counseling	Prospect theory and gain-framed messaging		11/7	
Naito et al [82]	Duration (weeks): 8; partial supervision	Objective	WAT	Exercise goal or program and in-person counseling	None		12/7	
Befort et al [83]	Duration (weeks): 26; no supervision	Subjective	WAT and phone	Exercise goal or program, PA log, phone counseling, group interaction, and DVD	SCT		13/9	
Nápoles et al [84]	Duration (weeks): 8; no supervision	Objective	WAT, mobile app, and phone	Print materials and phone counseling	SCT		11/7	
Pope et al [85]	Duration (weeks): 10; no supervision	Objective	Mobile app	Group interaction	SCT		9/6	
Spark et al [86]	Duration (weeks): 26; follow-up (weeks): 52; no supervision	Objective	SMS text messaging	Phone counseling	None		15/7	
Wilson et al [87]	Duration (weeks): 8; partial supervision	Objective	WAT	Exercise goal or program and group interaction	Health belief model		9/7	
Chung et al [88]	Duration (weeks): 6; no supervision	Objective	Mobile app	PA log and group interaction	None		5/5	
Nyrop et al [89]	Duration (weeks): 12; no supervision	Subjective and objective	Website and WAT	PA log and print materials	None		5/5	
Cairo et al [90]	Duration (weeks): 24; no supervision	Subjective	SMS text messaging and mobile app	Print materials and DVD	None		5/5	
Cheong et al [91]	Duration (weeks): 12; no supervision	Subjective	WAT and mobile app	Exercise goal or program	None		16/10	
Groen et al [92]	Duration (weeks): 16; no supervision	Subjective	Website	None	None		10/6	
Hong et al [93]	Duration (weeks): 10; no supervision	Subjective	Website	None	Goal-setting theory		—^j^	
McCarroll et al [94]	Duration (weeks): 4; no supervision	Subjective	Mobile app	None	SCT		13/8	
MacDonald et al [95]	Duration (weeks): 8; follow-up (weeks): 20; no supervision	Subjective	Website, WAT, mobile app, and phone	Exercise goal or program and phone counseling	Motivational interviewing and cognitive behavioral therapy		42/12	
Gell et al [96]	Duration (weeks): 4; partial supervision	Objective	Website, WAT, SMS text messaging, and phone	Phone counseling and in-person counseling	SCT		14/8	
Puszkiewicz et al [97]	Duration (weeks): 6; no supervision	Subjective	Mobile app	None	None		14/10	
Short et al [98]	Duration (weeks): 2; partial supervision	Subjective	Email and mobile app	Phone counseling and in-person counseling	None		9/6	
Abbott et al [99]	Duration (weeks): 12; partial supervision	Subjective	WAT and SMS text messaging	PA log, print materials, and in-person counseling	Gain-framed messaging		12/9	
Javaheri et al [100]	Duration (weeks): 4; partial supervision	Objective	WAT and phone	Exercise goal or program, PA log, print materials, phone counseling, and in-person counseling	None		9/6	
Zhang et al [101]	Duration (weeks): 26; partial supervision	Subjective and objective	Website, WAT, and phone	Exercise goal or program, phone counseling, group interaction, and DVD	None		8/7	
Trinh et al [102]	Duration (weeks): 12; follow-up (weeks): 24; partial supervision	Objective	Website and WAT	None	None		14/8	

^a^Studies were sorted by study type, cancer type, and treatment. The follow-up duration is listed as total duration in weeks from baseline. Behavior change techniques (BCTs) are listed as the total number of BCTs and the number of BCT categories covered.

^b^PA: physical activity.

^c^BCT: behavior change technique.

^d^WAT: wearable activity tracker.

^e^HAPA: health action process approach.

^f^SDT: social determination theory.

^g^SCT: social cognitive theory.

^h^TPB: theory of planned behavior.

^i^TTM: transtheoretical model.

^j^Did not provide sufficient details to code BCTs.

### Current State of the Literature

#### Study Characteristics

Studies were conducted in 8 different countries: United States (34/67, 51%), Australia (9/67, 13%), Canada (7/67, 10%), South Korea (7/67, 10%), The Netherlands (5/67, 8%), the United Kingdom (3/67, 5%), Japan (1/67, 2%), and France (1/67, 2%). Almost 50% of the articles (32/67, 48%) were published after July 2018 (Figure S1, Multimedia Appendix 1).

#### Participant Characteristics

A total of 6655 participants were enrolled across 67 studies with a median sample size of 51 (range 10-492). Participants were, on average, 56.7 (SD 8.2) years old. Approximately one in 3 studies recruited breast cancer survivors (24/67, 38%) or included multiple cancer types (23/67, 34%); 57% (38/67) of studies including only those who had completed treatment. Ethnicity was reported in 60% (40/67) of the studies, and 79.2% (SD 28.1%) of the participants were Caucasian. Only 9% (6/67) of the studies intentionally recruited non-Caucasian participants.

#### Study or Intervention Design

Approximately 67% (45/67) of studies used randomized trial designs with ≥2 study groups, whereas the remaining 33% (22/67) were nonrandomized single or two-arm trials. Across studies, the duration ranged from 1-52 weeks, with a median of 12 weeks. A total of 12 (18%) studies reported outcomes at a follow-up time point to assess the maintenance of intervention effects. Although all articles listed PA as an objective, their primary objectives varied widely. PA was the primary outcome of interest in 43% (29/67) of the studies. Other primary outcomes included feasibility (26/67, 39%), physical function (5/67, 8%), psychosocial function (4/67, 6%), and fatigue (3/67, 5%).

All the described interventions were either partially supervised (18/67, 27%), with both in-person and unsupervised components, or fully unsupervised (49/67, 72%). The interventions used between one and five technology components, with two (27/67, 40%) being the most common. Wearable devices (41/67, 61%) and websites (32/67, 48%) were the most frequently used technology components for delivering intervention content. Other common technology components used were SMS text messages (19/67, 28%), mobile apps (18/67, 27%), and email (15/67, 22%). Telephone contact was used in 37% (25/67) of the interventions. Figure S2 in Multimedia Appendix 1 presents the trends in eHealth used in the included studies over time. A specific exercise program or prescription was provided in 37% (25/67) of the studies, whereas PA logs were used in 28% (19/67). Instructions via print materials (16/67, 24%) and DVD (7/67, 10%) were less common. Finally, many studies provided additional interaction via phone counseling (25/67, 37%), in-person counseling (16/67, 24%), or group-based formats (16/67, 24%).

#### Use of Theory and BCTs

More than one-third of the trials (26/67, 39%) did not report using behavioral theories to guide intervention design. Of the remaining studies, 34% (23/67) used social cognitive theory, 9% (6/67) used the transtheoretical model, and 9% (6/67) used the theory of planned behavior, whereas various other theories were applied in 25% (17/67) of studies [103-105].

With respect to BCTs, across all studies, 69% (64/93) BCTs (covering 15 of 16 categories) were implemented at least once [25]. The number of techniques applied ranged from 5-42, across 2-14 categories of the behavior change taxonomy, with 9 (8/67, 12%) being the most common. The frequency of use of the most common BCTs and all behavior change categories used are displayed in Figure S3 of Multimedia Appendix 1. The four techniques (*self-monitoring of behavior*, *credible source*, *goal-setting of behavior*, and *adding objects to the environment*) and four categories (*goals and planning*, *feedback and monitoring*, *antecedents*, and *comparison of outcomes*) were found in >90% of the studies. In contrast, the prevalence of four categories (*regulation*, *scheduled consequences*, *covert learning*, and *identity*) was <10%.

#### PA Outcomes

The measurement of PA was highly variable across studies. Subjective PA measures were used in 45% (30/67) of the studies, whereas 33% (22/67) used objective measures, and the remaining 22% (15/67) used both. The subjective PA questionnaires used were the Godin Leisure Time Exercise Questionnaire (16/67, 24%), International PA Questionnaire (10/67, 15%), as well as 17 other questionnaires (19/67, 28%) [106,107]. Accelerometers and pedometers were used to measure PA objectively in 39% (26/67) and 10% (7/67) of the studies, respectively. These included both research-grade and commercial sensors.

**Figure 2:**
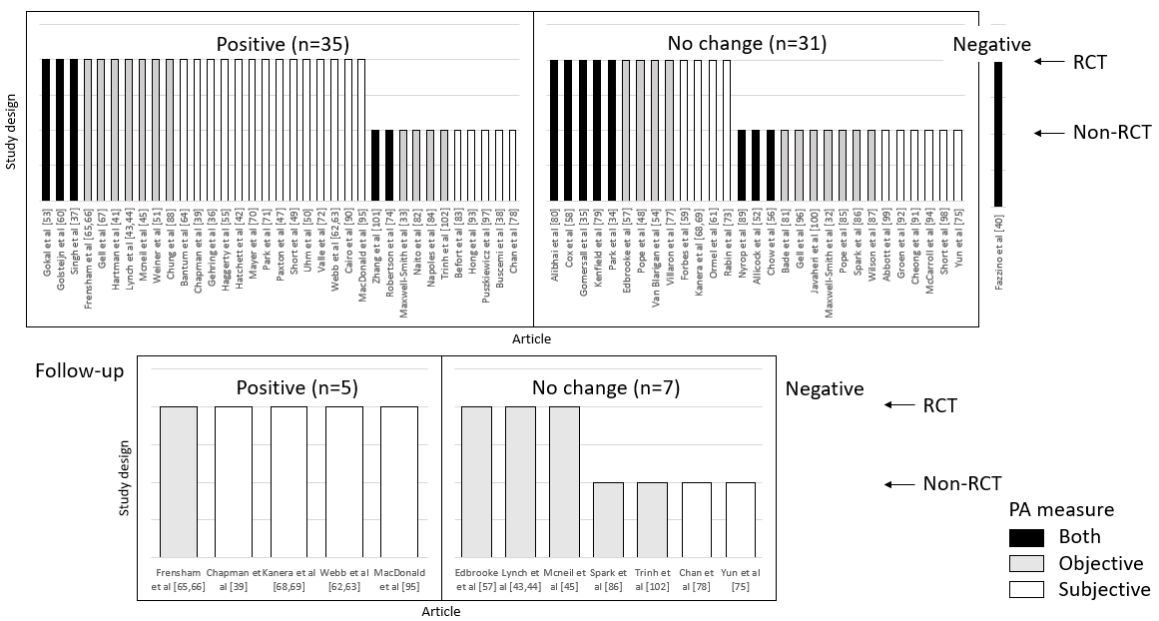
Harvest plots for physical activity outcomes. Studies were grouped according to the statistical significance of their physical activity outcomes (physical activity increase, physical activity decrease, or no change). Bar height distinguishes between randomized controlled trials (high) and other study designs (low). Shading specifies how physical activity was measured (subjective, objective, or both). PA: physical activity; RCT: randomized controlled trial.

As seen in Figure 2, statistically significant postintervention improvements in PA behavior were reported in 52% (35/67; 18 between-group, 17 within-group) of interventions. The remaining 32 interventions reported in no change (29/67, 43%), decreases in PA (1/67, 2%), or did not report on statistical significance (2/67, 3%). Studies that found statistically significant changes in PA, as well as those that did not, included participants with mixed cancer types, stages, and treatment status. The only intervention where PA decreased significantly was a 52-week RCT for patients with off-treatment breast cancer [40]. Only 18% (12/67) of interventions tracked participants beyond the intervention (ie, between 12 and 52 weeks postintervention) to assess PA maintenance. Significant improvements in PA behavior were measured in 42% (5/67; 4 measured significant improvements directly postintervention) of the studies at the follow-up assessment (Figure 2). The remaining 58% (7/67; 4 measured significant improvements directly postintervention) of the studies reported no change.

### Intervention Characteristics That May Promote PA Behavior Change: Weight Analysis

#### Primary Outcomes and Supervision

**Figure 3:**
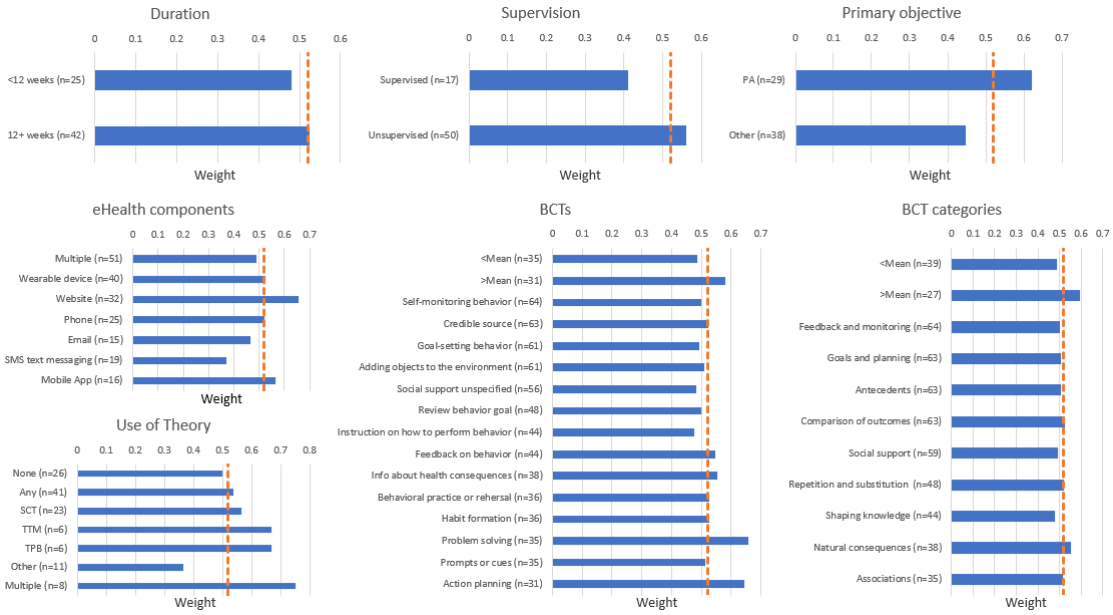
Weight analyses grouped by intervention characteristics. The orange dotted line represents the weight of significant changes in physical activity levels across all 67 studies (0.522). BCT: behavior change technique; PA: physical activity; SCT: social cognitive theory; TPB: theory of planned behavior; TTM: transtheoretical model.

The results of the weight analyses, which were used to explore associations between intervention elements and PA outcomes, are presented in Figure 3. Studies with PA as the primary outcome (29/67, 43%) had a weight of 0.621, compared with 0.447 when PA was a secondary outcome (38/67, 57%). Interventions that were unsupervised (ie, no in-person elements during the intervention period; 50/67, 75%) had a weight of 0.560, whereas those with some supervision (17/67, 25%) had a weight of 0.412.

#### eHealth Components

When a wearable device (40/67, 60%) or app (16/67, 24%) was used in an intervention, the weights were 0.525 and 0.563, respectively, as compared with a weight of 0.522 across all 67 studies. The use of websites as part of the intervention was associated with a weight of 0.656 (32/67, 48%), whereas SMS text messaging (0.368; 19/67, 28%), email (0.467; 15/67, 22%), and the use of multiple technologies (0.490; 51/67, 76%) had lower weights.

#### Use of Theory

The use of any behavioral theory in an intervention (41/67, 61%) was associated with a weight of 0.528, whereas interventions that did not report the use of theory (26/67, 39%) had a weight of 0.500. The most common theories, social cognitive theory (23/67, 34%; 0.565), transtheoretical model (6/67, 9%; 0.667), and theory of planned behavior (6/67, 9%; 0.667), were all associated with weights >0.522 [103-105]. When multiple theories were used in a single intervention (8/67, 12%), the weight increased to 0.750. The weights for other theories were not calculated because of the small number of studies using each one.

#### Behavior Change Techniques

The weight of 46% (31/67) of the interventions that incorporated more than the mean number of 13.5 BCTs was 0.581, whereas the weight of the 52% (35/67) of the interventions that used less than 13.5 BCTs was 0.486. Among the 14 BCTs used in at least 45% of the interventions, *problem solving* (0.657; 35/67, 52%) and *action planning* (0.645; 31/67, 46%) had the highest weights. The remaining weights ranged from 0.477-0.553 (Figure 3). Of the nine BCT categories coded in ≥50% of the interventions, category 5 *natural consequences* (0.553; 38/67, 57%) and category 9 *comparison of outcomes* (0.524; 63/67, 94%) were associated with the highest weights.

### RoB and Completeness of Reporting

The overall RoB among the 45 RCTs ranged from some risk (4/45, 8%) to high risk (41/45, 91%). This was largely because of RoB in deviation from the intended intervention (7/45, 15% some risk; 38/45, 84% high risk) and measurement of the outcome (31/45, 68% high risk). Most studies had a low RoB for the remaining categories (n=34-44, depending on the category). Because of the risk of confounding, 95% (21/22) of the nonrandomized studies were found to have critical RoB. RoB in the measurement of outcome was moderate (10/67, 15%) or serious (9/67, 13%) for most single-arm studies, whereas it remained low across other categories (see Figure S4 in Multimedia Appendix 1 for RoB among the included studies [32-102]). If not for the lack of blinding, then only 58% of studies would have had a high overall high RoB, mainly because of bias in outcome measurement owing to the reliance on self-reported PA. Mean completeness of reporting was moderate, with 69.4% (71.4% for RCTs and 65.2% for nonrandomized studies) of applicable CONSORT-eHealth items covered in the included publications. Nearly one-third of the applicable items (mean of 30.6%, SD 9.4%) were not reported. For RCTs and nonrandomized studies, mean values of 15.5% (SD 3.4%) and 32.4% (SD 4.7%), respectively, of CONSORT-eHealth items (overall mean 20.8%, SD 8.8%) were not applicable on a case-by-case basis.

## Discussion

### Principal Findings

The purpose of this review was to provide a comprehensive, updated overview of eHealth intervention research designed to promote PA and to explore intervention characteristics (ie, duration, delivery modalities, use of theory, and BCTs) associated with increased PA levels. Many of the included studies were published after July 2018 and focused on feasibility, which indicates the rapidly growing yet early state of the field. Across the studies, there was substantial heterogeneity in the participants, interventions, and outcomes. All studies had high RoB for some domains, and incomplete reporting was problematic. Nevertheless, findings suggest that eHealth may be an effective strategy to enhance PA levels with selected modalities, BCTs, and behavioral theories that potentially enhance effectiveness.

### Current State of the Literature

The growing number of published articles reporting on eHealth PA interventions for adults with cancer (48% of articles published since July 2018) aligns with several funding calls for eHealth research, institutional strategic priorities, and the growing prevalence of, and preference for, eHealth among adults with cancer [12-14]. With the restrictions imposed by the COVID-19 pandemic on face-to-face PA programs, continued acceleration in this field is expected [108]. The COVID-19 pandemic has highlighted the need for eHealth PA interventions in oncology, and such interventions will continue to remain relevant beyond the pandemic, especially for improving the reach of PA interventions to underserved populations with cancer (eg, remote or rural) [8,108]. For example, an ongoing study in Canada that aims to bring exercise oncology programs to remote and rural cancer populations has delivered all classes remotely during the COVID-19 pandemic and will continue to offer videoconference-based programs (NCT04478851) [109,110]. As many of the included studies tested the feasibility of using eHealth for PA promotion in adults with cancer (36%) using single-arm designs or smaller RCTs, the findings on the effectiveness to change PA levels remain largely preliminary. Next steps could include study designs, such as factorial RCTs or alternative trial designs with the capacity to quantify the contribution of intervention effectiveness from various technology components, theories, and BCTs. Finally, larger multisite RCTs or meta-analyses of comparable studies to strengthen the evidence for the effectiveness of these interventions will be required to continue to grow our knowledge [111-113].

Overall, this review highlights that eHealth interventions can increase PA levels, with 52% of the studies reporting significant increases in postintervention PA. Previous reviews have reported that 50%-80% of eHealth PA interventions for adults with cancer reported significant improvements in PA levels [15-19]. Differences in these findings maybe because of the inclusion of studies that were underpowered to detect changes in PA levels (ie, feasibility trials and those aiming to impact a primary outcome other than PA levels), as well as intervention heterogeneity (ie, varied duration, delivery modalities, use of theory, and BCTs). Nevertheless, eHealth PA interventions have the potential to enhance PA levels, although optimization is required. The first step to optimization is to examine eHealth PA intervention components and their impact on effectiveness to change PA behavior.

### Intervention Characteristics That May Promote PA Behavior Change

Findings from this review show that both well-established eHealth components (eg, informational websites) and emerging technologies (eg, mHealth) were associated with increased PA levels both when used alone or in combination with other eHealth. Researchers are encouraged to consider the pros and cons for each type of eHealth when designing eHealth PA interventions. For example, the pros of mHealth include the ability to deliver real-time, context-aware behavior change interventions; passively monitor PA; and relative ubiquity in developed countries (eg, nearly 90% of Canadians own a smartphone) [11,114,115]. Meanwhile, websites that have the highest weight of any eHealth component may be selected for their familiarity and ease of use among older adults [116]. Moving forward, remaining flexible to align eHealth interventions with participant needs and preferences will likely be important [117,118].

A finding from this review that stands in contrast to those of previous reviews in exercise oncology is that a higher percentage of unsupervised interventions (56%; those without face-to-face interaction) were successful at increasing PA levels compared with those that were partially supervised (41%; those with one or more face-to-face components) [7,119]. This may be because of feelings of autonomy promoted by unsupervised interventions, a factor that has been linked to increased intrinsic motivation and PA behavior change [120-122]. In addition, it may be in part because of the more frequent use of behavioral theories (unsupervised: 63%; supervised: 56%) and BCTs (unsupervised mean: 13.8; supervised mean: 11.8) in the included unsupervised interventions, which have been associated with effectiveness in web-based behavioral interventions [123]. Direct comparisons of unsupervised and partially supervised eHealth PA interventions will be required to draw definitive conclusions on their relative effectiveness.

Recommendations have been made to use behavioral theories to guide intervention design to enhance the effectiveness of behavior change interventions [21,22]. Common behavioral theories, such as social cognitive theory, the transtheoretical model, and the theory of planned behavior, have been used in roughly half of eHealth PA interventions for adults with cancer [103-105]. Although the weights for studies using social cognitive theory, the transtheoretical model, the theory of planned behavior, or multiple theories (0.565-0.750) were higher than of those using none at all (0.500), 50% of the interventions that were not theory based also resulted in significant increases in PA levels. Furthermore, it is possible that some articles may have drawn upon theoretically based intervention components without explicitly discussing the use of theory. These mixed results add to the ongoing debate on the role of behavioral theories in real-world interventions [124]. Further examination of the use of theory (eg, theoretical integration and/or use of technology-specific models or theories) is needed to understand its impact, or lack thereof, in eHealth PA interventions.

The most commonly used BCTs in this review of eHealth PA interventions were goal setting and self-monitoring, which is similar to what has been reported in face-to-face PA interventions [20]. However, more BCTs were used across studies in this review, for both mean number per study and overall variety, than in reviews assessing face-to-face interventions [20]. Notably, current findings align with earlier research that has also suggested that certain BCTs may be more effective than others [20,125,126]. Further research is needed to understand the use of BCTs (ie, types and combinations) and their potential impact on intervention effectiveness in eHealth PA research. Indeed, these weight analyses revealed that eHealth interventions with more BCTs were more likely to report significant improvements in PA levels.

### RoB and Completeness of Reporting

Most reviewed studies (93%) had high overall RoB (ie, in one or more domains). This was, in large part, because of the lack of blinding. The inability to blind participants and researchers to PA interventions is a commonly reported limitation, irrespective of eHealth use [18,127]. Consequently, if this domain were removed, then the RoB would remain high in only 58% of the studies, primarily because of the reliance on self-reported PA outcomes [128]. Where possible, researchers may wish to integrate both objective and subjective PA measures into studies to reduce RoB [128]. Objective PA assessment is increasingly accessible, given the activity trackers in mHealth (eg, phones) and decreasing costs. Finally, the finding that all included studies were incompletely reported is problematic. Researchers are urged to follow the reporting guidelines appropriate for their study design, which can be found on the web [129].

### Limitations

There are important considerations to keep in mind when interpreting the findings. The broad inclusion criteria of the review, although selected intentionally to provide a comprehensive overview of this emerging field, hindered the ability to perform quantitative meta-analyses. Despite the systematic review, additional articles may have been missed if published in gray literature or in other languages. Although weight analyses were performed to provide insights for future research, their outputs must be interpreted with caution, as they are not a measure of statistical significance. Any reported associations remain purely exploratory and must be substantiated in future robust study designs. In addition, more than half of the included studies were underpowered to detect changes in PA as a secondary outcome, which is likely to bias weights toward the null. Some study characteristics in the weight analyses were represented in only a few studies, and most studies used complex interventions, making it difficult to identify the effect of individual components on outcomes. Finally, the authors did not complete BCT coder training before extraction, which may have led to some inaccuracies in BCT coding. However, efforts were made to minimize errors by double checking all codes and discussing with the senior author (NCR), an expert in PA behavior change, as needed.

### Research Needs and Opportunities

Consolidating the evidence on eHealth PA interventions for adults with cancer led to the identification of several research needs and opportunities that remain to be addressed. First, only 9 studies featured follow-up assessments to track PA behavior change after intervention completion. Examining the long-term maintenance of PA is critical to determine whether these interventions can have a lasting impact on PA levels. Second, it will be important to explore whether completely unsupervised eHealth interventions or eHealth interventions with limited supervision can rival the effectiveness of face-to-face supervised PA programs to increase PA levels in adults with cancer. Such work is needed to advocate for eHealth use in this field and may be crucial to the implementation of scalable PA programs for adults with cancer. Third, examining the effectiveness of videoconferencing platforms, which have surged in popularity during the COVID-19 pandemic, is warranted. Videoconferencing has the potential to leverage the advantages of supervised interventions (eg, live tailored feedback, social interaction, and accountability) while remaining accessible [108]. Fourth, given the rapidly evolving nature of eHealth, testing effectiveness using fully powered alternative trial designs (eg, SMART [sequential multiple assignment randomized trial], microrandomized trials, and factorial RCTs) is warranted so that evaluation can better match the pace of development, heighten external validity, and inform the translation of evidence to practice [112,113]. Such designs also allow researchers to establish definitive links between intervention components and changes in PA levels, allowing for systematic optimization of effectiveness. Finally, evaluations of cost-effectiveness are needed to inform real-world implementations of eHealth PA behavior change programs, as none were reported herein [130].

### Conclusions

This review summarizes findings from the rapidly growing field of eHealth PA interventions for adults affected by cancer. Although eHealth use in these interventions varies widely, the results are suggestive of positive outcomes. Furthermore, most studies integrated BCTs and relevant theories. Efforts are required to understand eHealth PA interventions better by exploring the impact on PA maintenance, investigating ways to optimize their effectiveness (by using BCTs, theories, and emerging technologies), and affirming effectiveness by applying well-powered alternative trial designs. Despite the early and evolving nature of this field, positive results suggest there is a case for integrating eHealth with efforts to promote PA, health, and well-being for adults affected by cancer.

## Appendix

Multimedia Appendix 1 Additional information on the review methodology and the included studies.

## Abbreviations

BCT: behavior change technique
CONSORT: Consolidated Standards of Reporting Trials
mHealth: mobile health
PA: physical activity
PRISMA: Preferred Reporting Items for Systematic Reviews and Meta-Analyses
PROSPERO: International Prospective Register of Systematic Reviews
RCT: randomized controlled trial
RoB: risk of bias
ROBINS-I: risk of bias in nonrandomized studies of interventions
SMART: sequential multiple assignment randomized trial
