# Supplementary material for: Current Evidence and Directions for Future Research in eHealth Physical Activity Interventions for Adults Affected by Cancer: Systematic Review
Source: JMIR Cancer. 2021 Sep 20;7(3):e28852. doi: 10.2196/28852 (PMC8491123; doi:10.2196/28852)
Supplement: Multimedia Appendix 1 [file cancer_v7i3e28852_app1.docx]

# A systematic review of eHealth physical activity interventions for adults affected by cancer: current evidence and directions for future research

## Multimedia Appendix 1

Table S1: Medline search strategy

| 1. exp Accelerometry/ |
| --- |
| 2. exp Smartphone/ |
| 3. exp Text Messaging/ |
| 4. exp Social Media/ |
| 5. exp Fitness Trackers/ |
| 6. exp Telemedicine/ |
| 7. exp Internet/ |
| 8. exp Mobile Applications/ |
| 9. exp Cell Phone/ |
| 10. (smartphone* or smart-phone* or mobile phone* or cell phone*).tw,kf. |
| 11. ((phone* or mobile* or cell-phone* or smartphone* or smart-phone*) adj2 app*).tw,kf. |
| 12. ((fitness or activit*) adj2 (track* or monitor*)).tw,kf. |
| 13. (accelerometer* or accelerometry or pedometer*).tw,kf. |
| 14. (digital health or electronic health or mobile health or ehealth or e-health or mhealth or m-health or telemedicine or telehealthcare).tw,kf. |
| 15. (text messag* or SMS or computer tailoring or online or online intervention* or internet or web-based).tw,kf. |
| 16. (fitbit or garmin* or vivofit or vivoactive or vivosmart or vivomove or vivoki or apple watch).tw,kf. |
| 17. exp Neoplasms/ |
| 18. exp Cancer Survivors/ |
| 19. (neoplasm* or cancer* or melanoma or leukemia or lymphoma or sarcoma or carcinoma).tw,kf. |
| 20. (cancer adj2 survivor*).tw,kf. |
| 21. exp Exercise/ |
| 22. exp Exercise Therapy/ |
| 23. exp Physical Fitness/ |
| 24. (exercis* or group fitness or fitness class*).tw,kf. |
| 25. physiotherap*.tw,kf. |
| 26. ((physical or resistance or weight or aerobic or personal) adj2 train*).tw,kf. |
| 27. exercise oncology.tw,kf. |
| 28. (physical adj2 (activit* or therap* or fitness)).tw,kf. |
| 29. 1 or 2 or 3 or 4 or 5 or 6 or 7 or 8 or 9 or 10 or 11 or 12 or 13 or 14 or 15 or 16 |
| 30. 17 or 18 or 19 or 20 |
| 31. 21 or 22 or 23 or 24 or 25 or 26 or 27 or 28 |
| 32. 29 and 30 and 31 |

Figure S1: Overview of publications by year


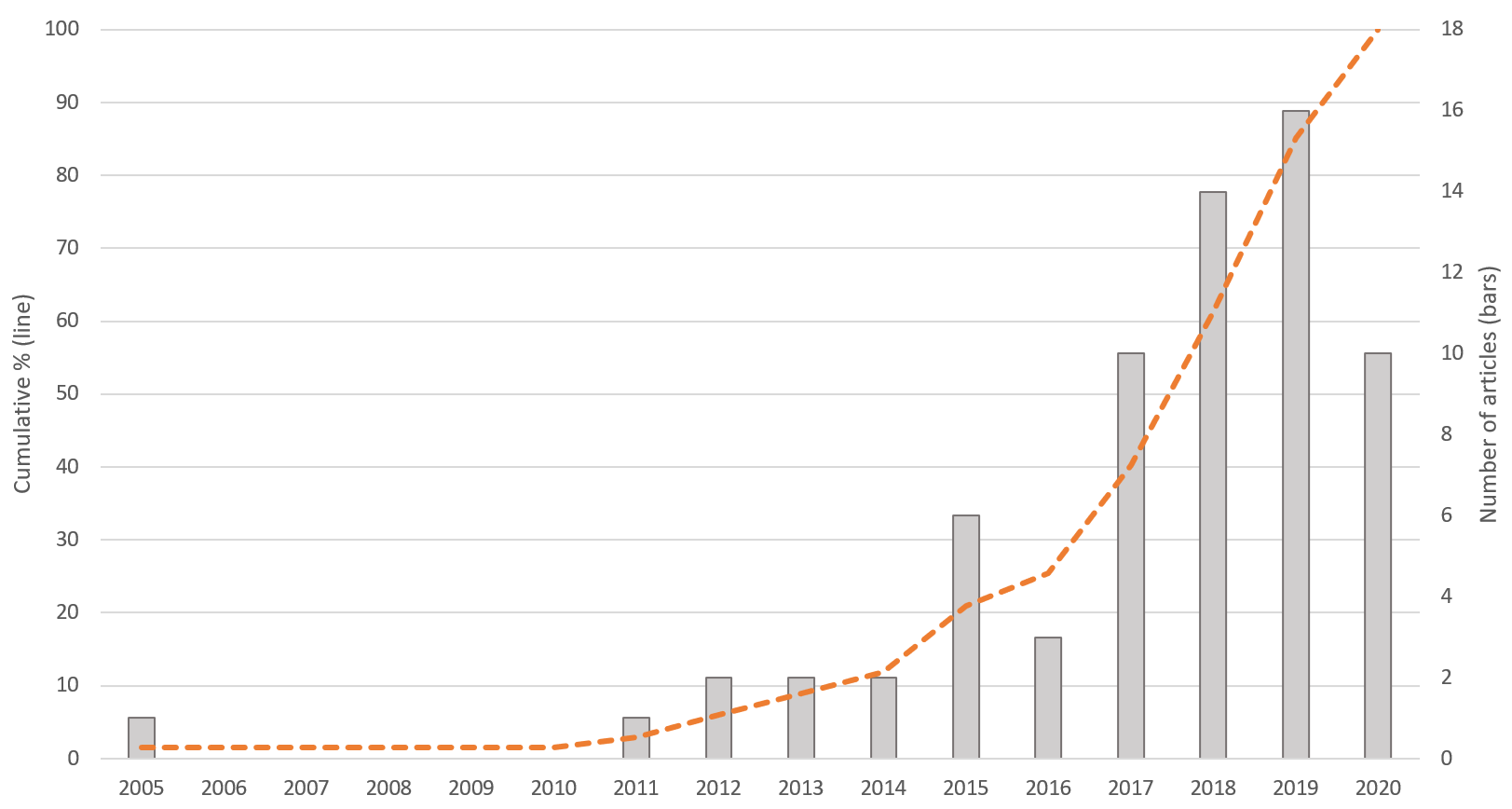


Figure S2: Trends in eHealth use by year


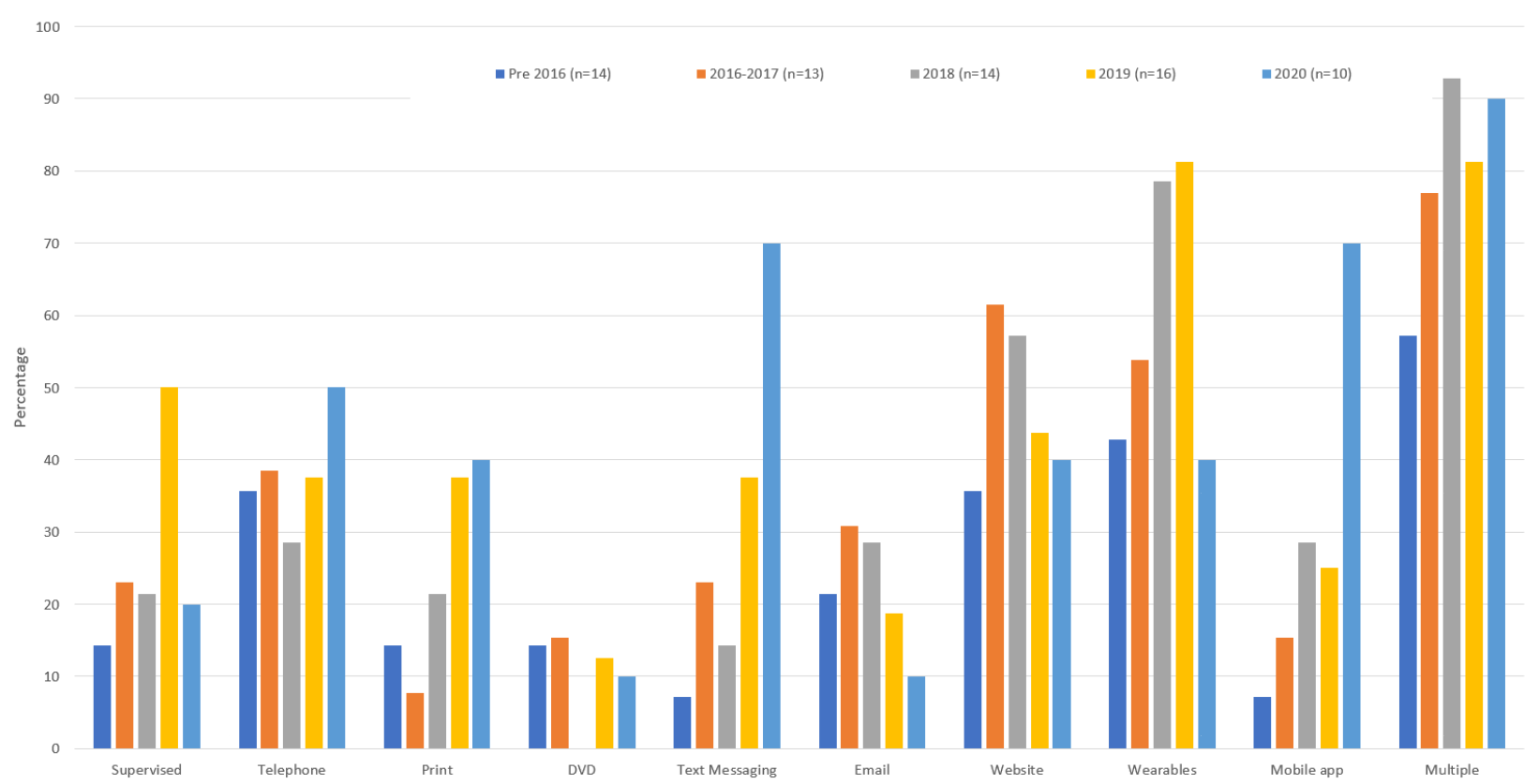


Figure S3: Frequency of use for most common behavior change techniques and categories


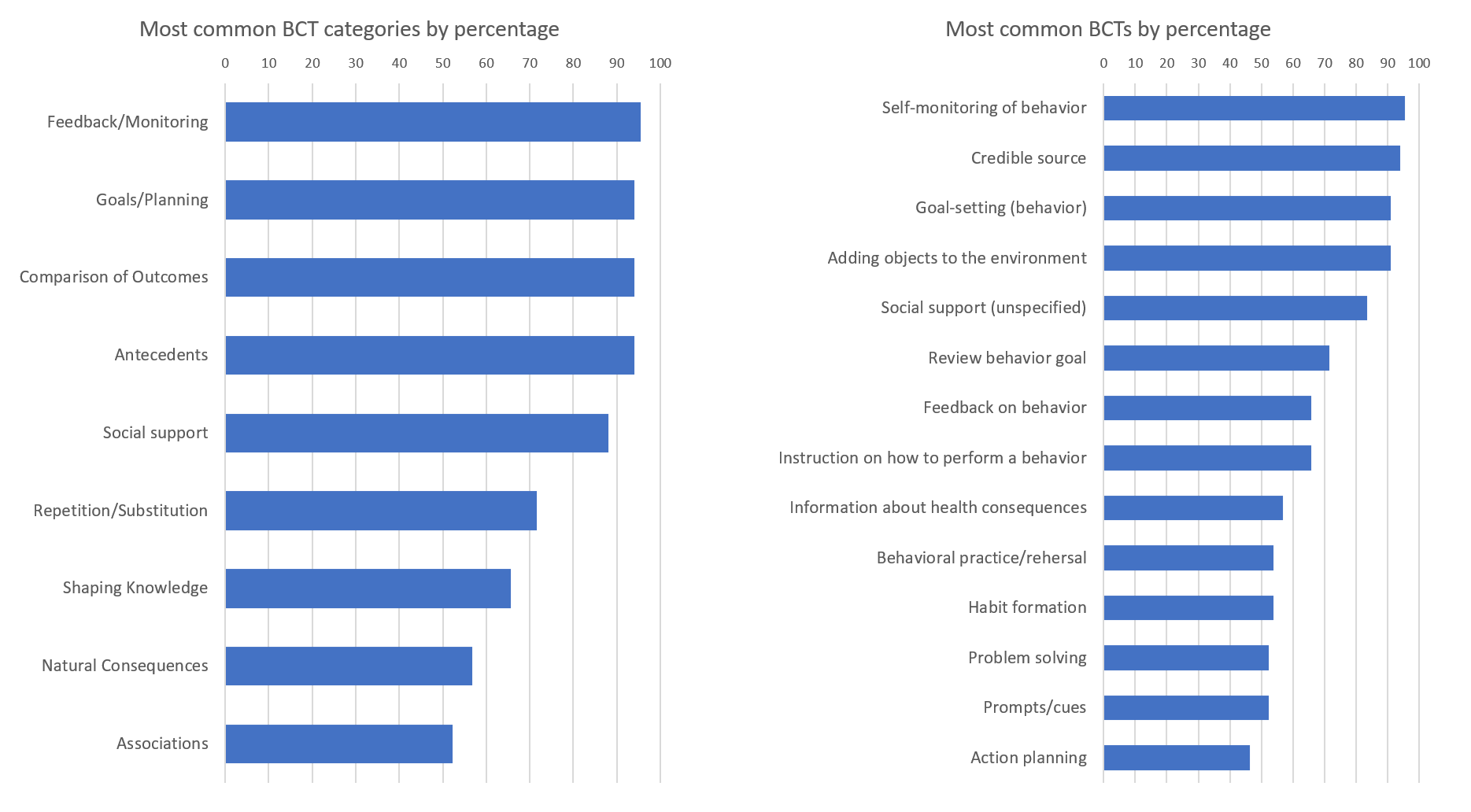


Figure S4: Risk of bias for individual studies


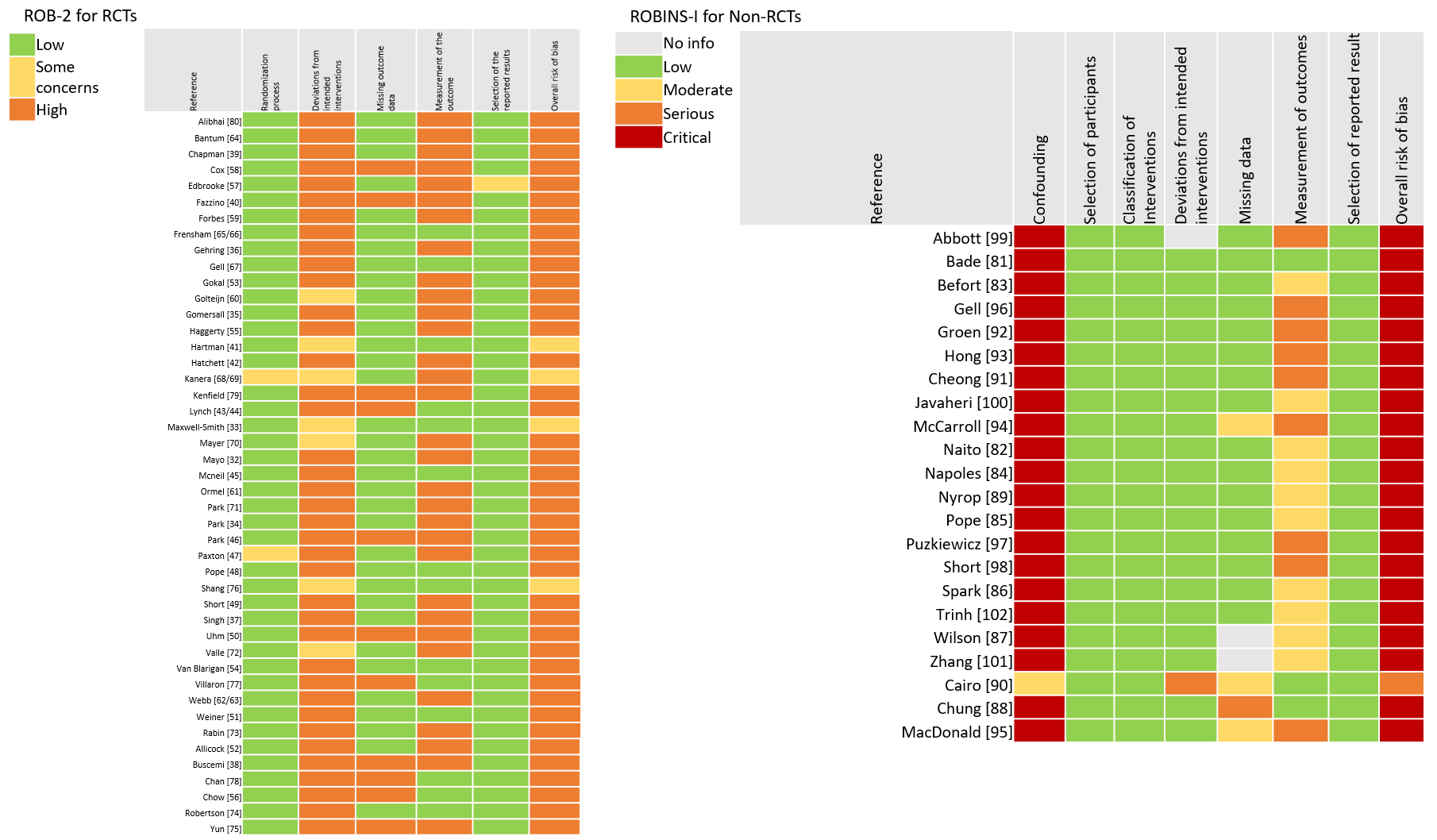


For full references, please refer to the corresponding reference number listed in the main article.
